# Positive Intraoperative Peritoneal Lavage Cytology is a Negative Prognostic Factor in Pancreatic Ductal Adenocarcinoma: A Retrospective Single-Center Study

**DOI:** 10.3389/fonc.2015.00182

**Published:** 2015-08-07

**Authors:** Kenichi Hirabayashi, Akiko Imoto, Misuzu Yamada, Atsuko Hadano, Nobuaki Kato, Youko Miyajima, Hitoshi Ito, Yoshiaki Kawaguchi, Toshio Nakagohri, Tetsuya Mine, Naoya Nakamura

**Affiliations:** ^1^Department of Pathology, Tokai University School of Medicine, Isehara, Japan; ^2^Department of Surgery, Tokai University School of Medicine, Isehara, Japan; ^3^Department of Gastroenterology and Hepatology, Tokai University School of Medicine, Isehara, Japan; ^4^Division of Diagnostic Pathology, Tokai University Hospital, Isehara, Japan

**Keywords:** cytology, peritoneal lavage, ascites, pancreatic cancer, adenocarcinoma, survival analysis

## Abstract

**Objective:**

The aim of this study is to evaluate the prognostic significance of intraoperative peritoneal lavage cytology (PLC) in pancreatic invasive ductal adenocarcinoma.

**Methods:**

Intraoperative PLC was evaluated in 162 patients with resectable pancreatic invasive ductal adenocarcinoma. The results were analyzed for correlations with clinicopathological parameters and/or prognoses.

**Results:**

In the 162 cases of resectable pancreatic ductal adenocarcinoma, 18 (11%), 141 (87%), and 3 (2%) were positive, negative, and equivocal for intraoperative PLC, respectively. Intraoperative PLC positivity was associated with older patients (over 65 years), large tumor size (over 35 mm), tumor location in the body/tail of the pancreas, and distant metastasis. Univariate analysis showed that larger tumor sizes (≥35 mm, *P* = 0.001), lymph node metastases (*P* = 0.005), distant metastasis (*P* = 0.004), advanced stage (stage IIB or III, *P* = 0.006), advanced tumor histological grade (G3, *P* < 0.001), or positive intraoperative PLC (*P* = 0.002) are associated with a shorter survival. Multivariate analysis revealed that larger tumor sizes (≥35 mm, *P* = 0.026), lymph node metastasis (*P* = 0.021), advanced tumor histological grade (G3, *P* < 0.001), and positive intraoperative PLC (*P* = 0.002) were independent prognostic factors.

**Conclusion:**

Intraoperative PLC is an independent prognostic factor for resectable pancreatic invasive ductal adenocarcinoma.

## Introduction

Pancreatic ductal adenocarcinoma is the most common pancreatic neoplasm, and is highly aggressive and malignant with a very low-survival rate. Several factors, such as large tumor size, retroperitoneal invasion, location of the tumor in the body or tail of the pancreas, and lymph node metastasis, are correlated with poor prognosis (1). Ascites or peritoneal lavage fluids are occasionally analyzed in patients of several cancer types to evaluate the peritoneal dissemination of tumor cells. In ovarian and fallopian tube cancers, the detection of tumor cells in ascites or peritoneal lavages is considered a negative prognostic factor, and peritoneal cytology was adopted as one of the factors determining the stage of the tumor by the International Federation of Gynecology and Obstetrics as well as the Union for International Cancer Control/American Joint Committee on Cancer (UICC/AJCC) classification system (2–4). In pancreatic ductal adenocarcinoma, the incidence of positive peritoneal cytology was 5–30% in resectable cases (5–14) and 20–57% in unresectable cases (6, 8, 9, 11, 15). However, the prognostic significance of positive peritoneal cytology has been controversial for pancreatic ductal adenocarcinoma. Some studies reported that positive peritoneal cytology is associated with poor prognosis (5, 6, 8–10, 13–17). In contrast, other studies reported that peritoneal cytology findings do not influence the patients’ prognosis (6–8, 11, 12, 18).

In this study, we retrospectively analyzed the relationship between intraoperative peritoneal lavage cytology (PLC) outcomes, clinicopathological parameters, and patient survival in resectable pancreatic ductal adenocarcinoma cases treated at Tokai University Hospital.

## Materials and Methods

### Cases

We retrospectively analyzed 162 consecutive cases of surgically resected, conventional pancreatic ductal adenocarcinoma with intraoperative PLC examination treated between 2006 and 2013 at Tokai University Hospital. All cases were diagnosed based on routine histological examination. We excluded cases of special histological types, such as adenosquamous carcinoma, mucinous carcinoma, undifferentiated carcinoma, and intraductal papillary mucinous neoplasms with invasion, as well as cases that had undergone preoperative chemotherapy. Tumor resectability was evaluated with computed tomography and at laparotomy. Lesions eligible for resection included those without distant metastases and those with or without involvement of the portal or superior mesenteric vein as long as resection and reconstruction margins were deemed safe. Furthermore, tumors with gastroduodenal artery encasement up to the hepatic artery, with either short segment encasement or direct abutment to the hepatic artery, were deemed resectable, as were lesions with no extensions to the celiac axis and tumors encasing the superior mesenteric artery in a manner not exceeding 180° of the circumference of the vessel wall (19). pT-stage, pN-stage, and tumor grade (G) were categorized according to the UICC/AJCC classification (3, 4). After laparotomy, the Douglas fossa was washed with 200–300 mL of saline, and 10 mL of peritoneal ascites was aspirated using a syringe. The collected peritoneal lavage fluid was centrifuged for 10 min at 600 × *g*. After discarding the supernatant, precipitated cellular components were smeared onto glass slides, fixed in 95% ethanol, and subjected to Papanicolaou staining and/or were dried and subjected to Giemsa staining. Cytological results were classified as negative, positive, or equivocal. The clinical data were collected from individual patient records; patients’ clinicopathological features are summarized in Table 1. This study was approved by the Research Ethics Committee of Tokai University School of Medicine (No. 14R116).

**Table 1:** **The clinicopathological features**.

**Total number of cases**	162
**Mean age, year (SD)**	67.2 (8.6)
**Sex, no. (%)**	
Male	77 (47)
Female	85 (53)
**Mean size of tumor, cm (SD)**	36.2 (16.6)
**Local invasion: pT, no. (%)**	
pT1	7 (4)
pT2	2 (1)
pT3	153 (95)
pT4	0
**Lymph node metastasis: pN, no. (%)**	
pN0	75 (46)
pN1	87 (54)
**Distant metastasis: pM, no. (%)**	
pM0	156 (96)
pM1	6 (4)
**Stage, no. (%)**	
IA	6 (4)
IB	2 (1)
IIA	67 (41)
IIB	81 (50)
III	0
IV	6 (4)
**Perineural invasion, no. (%)**	
Negative	10 (6)
Positive	152 (94)
**Lymphatic involvement, no. (%)**	
Negative	14 (9)
Positive	148 (91)
**Venous involvement, no. (%)**	
Negative	8 (5)
Positive	154 (95)
**Tumor site, no. (%)**	
Head	107 (66)
Body to tail	55 (34)
**Tumor grade, no. (%)**	
G1	61 (38)
G2	90 (55)
G3	11 (7)
**EUS-FNA cytology/biopsy**	
No	156 (96)
Yes	6 (4)
**Intraoperative peritoneal cytology, no. (%)**	
Negative	141 (87)
Positive	18 (11)
Equivocal	3 (2)

*EUS-FNA, endoscopic ultrasound-guided fine needle aspiration*.

### Statistical analysis

All statistical analyses were performed using SPSS version 19 (IBM Japan, Tokyo, Japan). For univariate analysis, Fisher’s exact test or Pearson’s chi-squared test was used to determine the relationship between clinicopathological features and PLC results. Multivariate analysis between clinicopathological features and PLC results was performed with logistic regression. Life-table probabilities for the overall survival were calculated using the Kaplan–Meier method, and the differences in survival between the subgroups were compared with the log-rank test. The Cox proportional hazard model was used to calculate the univariate and multivariate hazard ratios. A *P*-value of <0.05 was considered significant.

## Results

### Results of intraoperative PLC

Of 162 cases of resectable pancreatic ductal adenocarcinoma, 141 (87%) were negative for intraoperative PLC. Eighteen cases (11%) were positive, while three cases (2%) were equivocal (Table 1).

### Correlation between clinicopathological parameters and PLC

The correlation between PLC results and clinicopathological parameters was analyzed (Table 2). Of 159 cases examined after excluding 3 patients with equivocal cytology results, positive PLC was associated with tumor sizes 35 mm or larger (*P* = 0.01), ages 65 years and older (*P* = 0.01), tumor location in the body/tail of the pancreas (*P* = 0.047), and the occurrence of distant metastasis (*P* = 0.002).

**Table 2:** **Intraoperative peritoneal cytology and clinicopathological parameters**.

Total number of cases = 159	Intraoperative peritoneal cytology		*P*-value
	Negative (*n* = 141) *n* (%)	Positive (*n* = 18) *n* (%)	
**Age**
<65 years (*n* = 51)	50 (98)	1 (2)	0.01
≥65 years (*n* = 108)	91 (84)	17 (16)	
**Sex**
Male (*n* = 76)	66 (87)	10 (13)	0.484
Female (*n* = 83)	75 (90)	8 (10)	
**Tumor size**
<35 mm (*n* = 81)	77 (95)	4 (5)	0.01
≥35 mm (*n* = 78)	64 (82)	14 (18)	
**Local invasion: pT**
pT1 or 2 (*n* = 9)	9 (100)	0	0.599
pT3 or 4 (*n* = 150)	132 (88)	18 (12)	
**Lymph node metastasis: pN**
pN0 (*n* = 73)	64 (88)	9 (12)	0.712
pN1 (*n* = 86)	77 (90)	9 (10)	
**Distant metastasis: pM**
pM0 (*n* = 153)	139 (91)	14 (9)	0.002
pM1 (*n* = 6)	2 (33)	4 (67)	
**Stage**
Stage I or IIA (*n* = 73)	64 (88)	9 (12)	0.712
Stage IIB or III or IV (*n* = 86)	77 (90)	9 (10)	
**Perineural invasion**
Negative (*n* = 10)	10 (100)	0	0.605
Positive (*n* = 149)	131 (88)	18 (12)	
**Lymphatic involvement**
Negative (*n* = 14)	14 (100)	0	0.371
Positive (*n* = 145)	127 (88)	18 (12)	
**Venous involvement**
Negative (*n* = 7)	7 (100)	0	1
Positive (*n* = 152)	134 (88)	18 (12)	
**Tumor site**
Head (*n* = 104)	96 (92)	8 (8)	0.047
Body to tail (*n* = 55)	45 (82)	10 (18)	
**Tumor grade**
G1 or G2 (*n* = 148)	130 (88)	18 (12)	0.615
G3 (*n* = 11)	11 (100)	0	
**EUS-FNA cytology/biopsy**
No (*n* = 153)	137 (90)	16 (10)	0.138
Yes (*n* = 6)	4 (67)	2 (33)	

*EUS-FNA, endoscopic ultrasound-guided fine needle aspiration*.

### Survival analysis

**Figure 1:**
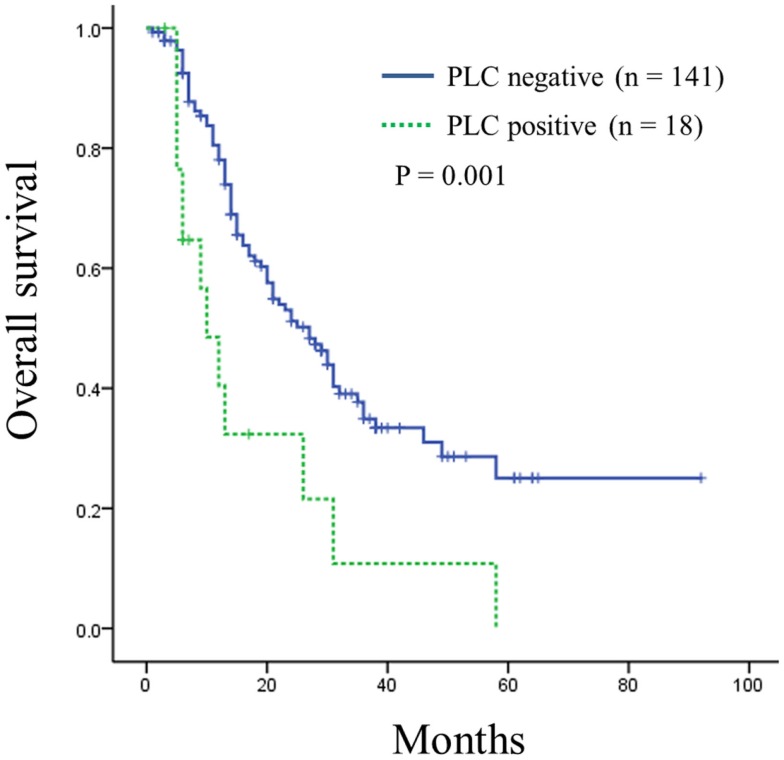
**Kaplan–Meier curve of overall survival in patients of resectable pancreatic ductal adenocarcinoma according to intraoperative peritoneal lavage cytology (PLC) status**. Survival was shorter for patients with positive PLC compared to those with negative PLC (*P* = 0.001).

Univariate analysis of overall survival in all pancreatic ductal adenocarcinoma patients revealed that cases significantly associated with shorter survival included those with larger tumor sizes (≥35 mm, *P* = 0.001), lymph node metastases (*P* = 0.005), distant metastasis (*P* = 0.004), advanced stage (stage IIB or III, *P* = 0.006), advanced tumor histological grade (G3, *P* < 0.001), or positive intraoperative PLC (*P* = 0.002) (Table 3). The median overall survival of patients with positive intraoperative PLC was significantly shorter than that of patients with negative PLC (10 vs. 27 months; *P* = 0.001) (Figure 1).

**Table 3:** **Univariate and multivariate analyses regarding the overall survival**.

	Univariate analysis		Multivariate analysis	
	HR (95% CI)	*P*-value	HR (95% CI)	*P*-value
**Age**
<65 years (*n* = 53)	1.004 (0.652–1.548)	0.984		
≥65 years (*n* = 109)	
**Sex**
Male (*n* = 77)	0.898 (0.596–1.353)	0.608		
Female (*n* = 85)	
**Tumor size**
<35 mm (*n* = 82)	2.046 (1.347–3.109)	0.001	1.639 (1.061–2.532)	0.026
≥35 mm (*n* = 80)	
**Local invasion: pT**
pT1 or 2 (*n* = 9)	2.483 (0.611–10.096)	0.204		
pT3 (*n* = 153)	
**Lymph node metastasis: pN**
pN0 (*n* = 75)	1.855 (1.205–2.855)	0.005	1.694 (1.083–2.652)	0.021
pN1 (*n* = 87)	
**Distant metastasis: pM**
pM0 (*n* = 156)	4.481 (1.601–12.541)	0.004		n.s.
pM1 (*n* = 6)	
**Stage**
Stage I or IIA (*n* = 75)	1.825 (1.186–2.808)	0.006		n.s.
Stage IIB or III (*n* = 87)	
**Perineural invasion**
Negative (*n* = 10)	2.04 (0.645–6.454)	0.225		
Positive (*n* = 152)	
**Lymphatic involvement**
Negative (*n* = 14)	2.305 (0.728–7.296)	0.155		
Positive (*n* = 148)	
**Venous involvement**
Negative (*n* = 8)	3.199 (0.786–13.023)	0.104		
Positive (*n* = 154)	
**Tumor site**
Head (*n* = 107)	0.964 (0.625–1.486)	0.867		
Body to tail (*n* = 55)	
**Tumor grade**
G1 or G2 (*n* = 151)	3.809 (1.949–7.444)	<0.001	4.411 (2.224–8.750)	<0.001
G3 (*n* = 11)	
**Intraoperative peritoneal cytology**
Negative (*n* = 141)	2.561 (1.414–4.638)	0.002	2.711 (1.464–5.022)	0.002
Positive (*n* = 18)	
**EUS-FNA cytology/biopsy**
No (*n* = 156)	1.317 (0.181–9.601)	0.786		
Yes (*n* = 6)	

*HR, hazard ratio; CI, confidence interval; n.s., not significant; EUS-FNA, endoscopic ultrasound-guided fine needle aspiration*.

Multivariate analysis indicated that larger tumor sizes (≥35 mm, *P* = 0.026), lymph node metastasis (*P* = 0.021), advanced tumor histological grade (G3, *P* < 0.001), and positive intraoperative PLC (*P* = 0.002) were independent prognostic factors for resectable pancreatic ductal adenocarcinoma (Table 3).

## Discussion

Some studies reported that positive ascitic cytology or PLC are positively correlated with lymph node metastasis, tumor location in the body/tail of the pancreas, larger tumor size, vascular invasion, serosal invasion, positive surgical margins, pT-factor, and advanced stage (5, 7, 9–14, 17). Our study revealed that positive intraoperative PLC in resectable pancreatic ductal adenocarcinoma was associated with larger tumor size (≥35 mm), older age (≥65 years), tumor location in the body/tail of the pancreas, and distant metastasis. Hence, our results are similar to those previously reported in the literature. As in previous studies, our data indicated that PLC positivity occurred in resectable pancreatic ductal adenocarcinomas in advanced stages.

The relationship between positive peritoneal cytology results and survival of resectable pancreatic ductal adenocarcinoma patients has been controversial. While several studies concluded that positive peritoneal cytology is associated with poor survival (5, 6, 9, 10, 13, 14), other studies reported no significant difference in prognosis between positive and negative peritoneal cytology (7, 8, 11, 12). Even with multivariate analysis, two conflicting results have been reported, one concluding that peritoneal cytology is an independent prognostic factor (14) and another stating that it is not (13).

The results of intraoperative PLC may be valuable for determining the subsequent treatment plan. Surgery for pancreatic cancer is highly invasive, and postoperative complications sometimes occur as well. Therefore, positive intraoperative PLC results may lead surgeons to decide to abandon the invasive resection procedure, particularly in patients who are older or in bad condition. Conversely, positive results in patients who are otherwise in good condition may prompt the pursuit of more aggressive postoperative therapy options.

In conclusion, this study revealed that positive intraoperative PLC in resectable pancreatic ductal adenocarcinoma was associated with older age, larger tumor size, tumor location in the body/tail of the pancreas, and distant metastasis. Patients with positive intraoperative PLC showed shorter survival times than those with negative PLC. Our data demonstrate that intraoperative positive PLC in resectable pancreatic ductal adenocarcinoma is a negative predictor of patient prognosis.

## Conflict of Interest Statement

The authors declare that the research was conducted in the absence of any commercial or financial relationships that could be construed as a potential conflict of interest.
